# Defensive medicine in dermatological practice – Dermatopathology as a mirror of structural challenges

**DOI:** 10.1111/ddg.15985

**Published:** 2025-12-24

**Authors:** Cornelia Sigrid Lissi Müller, Torsten Hansen, Claus Renzelmann

**Affiliations:** ^1^ Medical Care Center for Histology Cytology and Molecular Diagnostics Trier Germany; ^2^ Saarland University Faculty of Medicine Homburg/Saar Germany; ^3^ Law firm specializing in pathology law Wuppertal Germany

**Keywords:** Artificial intelligence (AI), defensive medicine, dermatopathology, diagnostic variability, medical liability, metacognition, overdiagnosis and underdiagnosis

## Abstract

Defensive medicine refers to diagnostic or therapeutic actions taken primarily to reduce legal liability rather than to benefit the patient. In dermatopathology, defensive practices manifest in frequent immunohistochemical testing, overly cautious report phrasing, and reliance on multidisciplinary tumor boards. Underlying causes include diagnostic uncertainty, guideline ambiguity, legal pressures, and financial incentives. These behaviors can lead to overtreatment and rising healthcare costs without improving diagnostic accuracy. This manuscript advocates for a balanced, evidence‐based diagnostic approach, emphasizing appropriate indications, improved clinicopathologic correlation, and the need for further subspecializing. Dermatopathology is presented as a paradigmatic discipline that highlights both the challenges and potential of defensive diagnostic behavior. Enhancing legal understanding, promoting structured decision‐making, and strengthening diagnostic confidence may help to reduce unnecessary testing and support high‐quality, patient‐centered care.

## INTRODUCTION AND DEFINITION

Modern medicine is increasingly shaped by a variety of external factors that influence clinical decision‐making. The traditional image of the unchallenged physician whose recommendations are followed without question has long ceased to reflect reality – if it ever did. Instead, medical practice today is markedly affected by legal uncertainties, economic pressures, and a growing tendency among patients to assert demands. These dynamics are particularly evident to younger physicians, who often find themselves making diagnostic decisions under structural constraints.^1^ In a recent position paper, the Ethics Section of the *German Interdisciplinary Association for Intensive and Emergency Medicine* (DIVI) underscores physicians’ autonomy in decision‐making as a core professional value. The authors highlight an increasingly consumer‐oriented attitude among patients, legal pressures and fears, and the progressive economization of medicine as key challenges. They call for stronger protection of professional autonomy to ensure patient care that is not only medically sound, but also economically reasonable and ethically justifiable.^1^ In this context, the concept of so‐called defensive medicine (DM), first described in the United States in the late 1960s, is a seamless extension of the discussion.^2^ A uniform definition has not yet been established by consensus; however, several publications in recent years have addressed this phenomenon and have attempted to translate the physicians’ “gut feeling” of unease into a descriptive framework.^3^ DM is currently defined, in most cases, by the various compensatory and reactive strategies available to physicians in a modern healthcare environment, and by their intent to avoid litigation. It refers to physicians’ efforts to protect themselves against legal or reputational consequences. A distinction is made between positive and negative DM, also referred to as “assurance and avoidance behavior.” Positive defensive medicine, or assurance behavior, includes additional medical services and excessive diagnostic testing or treatment. Negative DM, or avoidance behavior, involves the omission of potentially risky procedures/scenarios or the refusal to treat high‐risk cases – though the definition of “high‐risk cases” remains open.^4^, ^5^ Baungaard et al., in their systematic review, found that in 46% of the studies examined, DM represented a deviation from medical standards motivated by fear of medico‐legal claims.^3^

**FIGURE 1:**
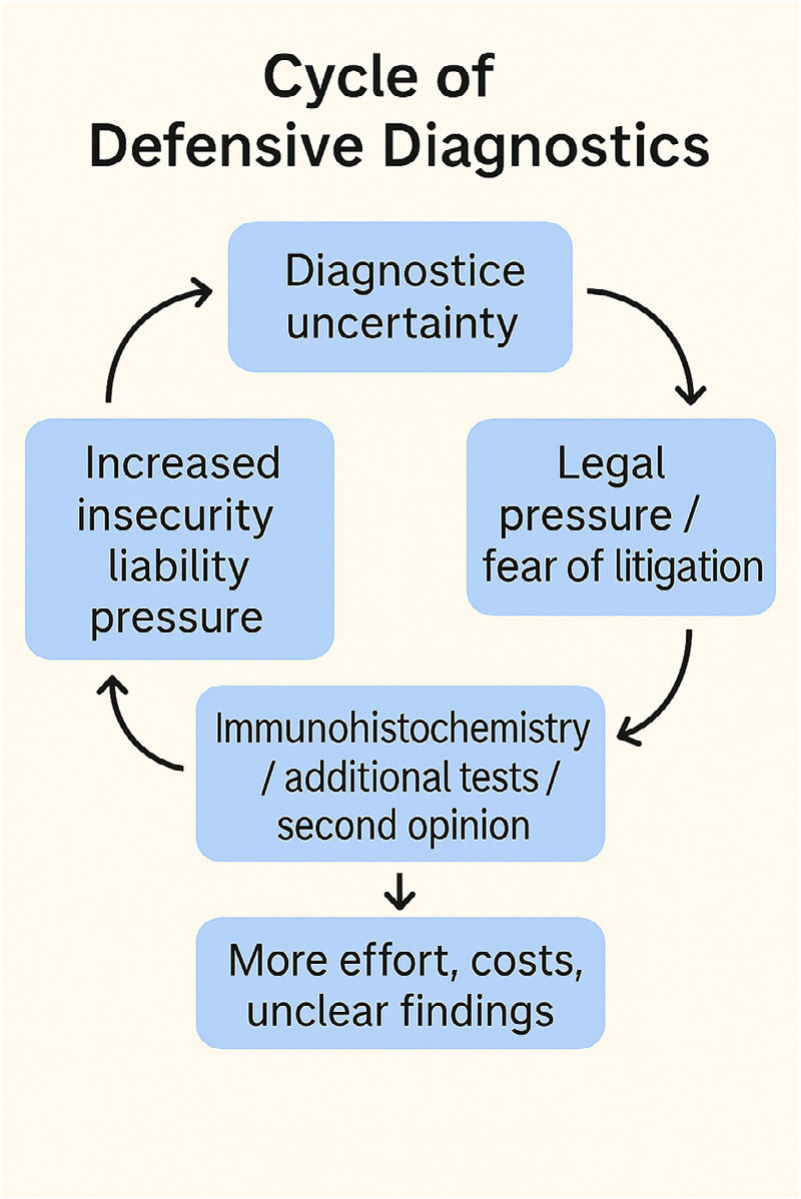
The illustration schematically represents the typical cycle of defensive diagnostic behavior in dermatopathology. It begins with diagnostic uncertainty, for example due to histological gray areas, interindividual variability, or missing clinical information. This uncertainty leads to legal pressure or fear of malpractice litigation. In response, additional immunohistochemical stains, molecular tests, or second opinions are often initiated – not always out of genuine diagnostic necessity, but as a means of legal protection. However, these measures do not necessarily improve diagnostic clarity; instead, they increase workload, costs, and sometimes even result in more ambiguous reports. This further amplifies the pathologist's sense of insecurity and liability – thus restarting the cycle. The graphic illustrates how well‐intentioned precautionary measures can evolve into a self‐perpetuating loop in which neither patient safety nor diagnostic quality automatically benefit.

The consequence of defensive medicine in practice – regardless of medical specialty – is a deprioritization of patient welfare. Defensive medicine primarily aims to prevent diagnostic errors. However, this approach does not necessarily lead to improved patient care. On the contrary, it often results in diagnostic ambiguities, neglect of complex clinical presentations, and increasing patient uncertainty due to a multitude of incidental findings without coherent clinical correlation. In addition to the desire to avoid legal consequences, reputational concerns – such as fear of conflict with patients, negative media coverage, or poor online reviews – also play a significant role (Figure 1).^3^

Baungaard et al. have summarized the consequences of DM clearly in their review. These include the problems of over‐ and underdiagnosis, increased healthcare costs, burdens on medical professionals, and ethical challenges. The authors understand “ethical challenges” to mean a disruption of the trustful relationship between physician and patient, as medical tests or procedures may be recommended primarily to safeguard the physician rather than to serve the diagnostic needs of the patient.^3^

### Defensive medicine in a societal context – causes, scope, and consequences

Defensive medicine is not an isolated phenomenon confined to individual medical specialties, but rather a manifestation of profound structural and societal developments in healthcare. It reflects a healthcare system that is increasingly regulated, commercialized, and legally controlled – where medical decision‐making operates under the primacy of risk aversion. This development is not merely the result of individual insecurity, but rather an expression of a broader cultural shift: the expectation of absolute error prevention, growing distrust toward professional expertise, and the expansion of what is medically feasible contribute to a climate of scrutiny and reassurance that increasingly puts pressure on clinical judgment.^6^, ^7^ Within this tension, defensive medicine does not emerge as an exception, but as a systematic behavioral pattern – legitimized by a safety‐oriented mindset that individualizes risk and increasingly defines responsibility in legal terms. In the process, the actual goal of medical practice – providing differentiated, context‐specific care aimed at the well‐being of the individual patient – fades into the background. Studies show that overdiagnostic strategies, such as excessive imaging, laboratory‐based marker analyses, or additional immunohistochemical testing, not only place a financial burden on the healthcare system but can also lead to unnecessary interventions and iatrogenic harm.^8^, ^9^

These tendencies can also be observed in dermatopathology. Immunohistochemical panels are often used for reassurance purposes, even when the clinical and histological diagnosis appears clear. In a large‐scale study on the assessment of melanocytic lesions, Titus et al. report that fear of legal vulnerability leads to an increase in avoidable additional testing – even though these tests are often diagnostically redundant.^10^ Although no systematic data are available, practical experience suggests that in a significant proportion of invasive melanomas, additional immunohistochemical studies are diagnostically unnecessary – estimates range from approximately 50% to 80%. A controlled comparison of histomorphologic findings before and after immunohistochemistry could help determine to what extent such supplementary studies truly contribute to the diagnostic outcome.

Consequently, it is problematic to consider guidelines or classifications as a blanket solution for defensive uncertainty. Even standardized directives – such as WHO criteria – can be overstretched for defensive purposes or interpreted as legal safeguards. They do not replace diagnostic judgment; they presuppose it. References to evidence‐based systems must therefore not be misunderstood as a justification for reassurance‐driven behavior, but must be critically complemented by the reflection that scientific standards themselves can be co‐opted defensively if the primary motivation is not knowledge acquisition but risk mitigation.^11^

This safety‐driven behavior is not exclusive to medicine but reflects a broader societal climate of increasing regulation and liability avoidance. As in other areas of life – such as building codes or traffic safety – unclear regulations in medical practice are often overinterpreted out of fear of sanctions, or pre‐emptively applied to situations where they provide no real added value. This “precaution by principle” can paradoxically result in the omission of meaningful measures while simultaneously encouraging the performance of unnecessary services. Defensive medicine thus emerges not only as an individual pattern of behavior but also as an institutional response to a culture of risk avoidance and externalization.

### The background noise of medical errors – the statistical inevitability of complications

Even under ideal conditions, medicine entails an irreducible residual risk of complications, as no diagnostic or therapeutic procedure can guarantee absolute safety.^12^, ^13^ The concept of such an irreducible residual risk – also referred to as the baseline complication rate or diagnostic background noise – points to unavoidable error rates, even in the presence of optimal technique and expertise. Interobserver variability and the inherent limitations of diagnostic methods are not exceptions but structural features of medical practice. However, the illusion of complete error‐free care fosters defensive behavior, such as excessive diagnostics performed for legal protection. A stronger incorporation of probabilistic reasoning – such as Bayesian logic – could help correct unrealistic expectations of safety in clinical practice.^14^

### Cross‐disciplinary evidence for defensive decision‐making

Defensive decision‐making is not an isolated phenomenon in dermatopathology but represents a widespread, cross‐professional response to an increasingly liability‐ and evaluation‐oriented healthcare system. Although this manuscript focuses on dermatopathological diagnostics, it must not be overlooked that defensive medical patterns can be observed across nearly all subspecialties of dermatology. In dermatosurgery, dermato‐oncologic therapy planning, and allergological diagnostics, decisions are increasingly shaped by considerations of legal liability and economics. For instance, in surgical practice, an international survey by Pietkiewicz et al. found that even the decision whether to use a new scalpel blade for each lesion during multiple excisions is not based solely on medical reasoning, but also made for legal protection – a subtle yet potentially significant expression of defensive practice.^15^ In oncology, decisions regarding systemic therapy in the face of unclear individual prognosis are frequently tied back to guideline recommendations – partly to ensure legal protection. This is especially true in borderline situations, where the data are ambiguous or the therapeutic benefit difficult to assess. Studies indicate that systemic therapies in advanced tumor stages are often continued even when no realistic chance of remission remains.^16^ While such practices are not necessarily defensive by definition, they exemplify how medical decision‐making is shaped by institutional, legal, or social conditions. In dermato‐oncology, for example, therapeutic indications are sometimes applied more generously – especially when a legally sound argument is desired due to clinical uncertainty. In addition to guidelines, interdisciplinary tumor boards play a key role: The consensus sought there may obscure existing uncertainties and distribute responsibility among many participants – an effect that may offer relief but still represents a form of defensive decision‐making.

This raises fundamental questions about the balance between medical necessity, patient‐centered indication, and liability‐driven risk avoidance – without calling into question the value of evidence‐based guidelines or multidisciplinary decision‐making. Welch and Black have shown, using oncology as an example, that a significant proportion of diagnosed cancers – particularly breast, prostate, lung, and thyroid cancers – would never have become symptomatic and thus constitute overdiagnosis. This form of diagnostic oversensitivity, often driven by legal and economic frameworks, not only imposes psychological and therapeutic burdens on patients but also presents an ethical challenge. The authors therefore call for a critical reflection on diagnostic standards and a renewed focus on true clinical relevance.^9^

Translating these findings to dermatology, it becomes evident that thresholds for histologic workup, surgical excision, or systemic therapy may also be lowered under internal or external pressures. Such developments should be examined from an interdisciplinary perspective and not be limited to the histological domain.^9^ Defensive behavior in allergology is often reflected in the excessive use of serological tests or skin test panels – even when the results have little or no influence on clinical decision‐making. In laboratory medicine, this phenomenon is well documented. Plebani reports that many excessively ordered tests are not medically indicated but are instead performed primarily to mitigate perceived liability risks.^17^ Although no specific allergological studies are available, the diagnostic environment is comparable: classical tests such as specific IgE, skin prick tests, or molecular allergy diagnostics are frequently used redundantly in practice – motivated by pressure to meet expectations, a desire for “completeness,” and fear of misdiagnosis. Similarly, in dermatological therapy, it has been shown that systemic antihistamines were prescribed in up to 77% of patients with atopic dermatitis – often despite a lack of evidence – driven more by a desire for diagnostic closure and reassurance.^18^

These examples emphasize the following: The issue of defensive medical decision‐making is not limited to histologic interpretation under the microscope but is a systemic phenomenon that permeates the entire spectrum of dermatologic care. A differentiated examination of this issue is essential in all subspecialties – both regarding care quality and the preservation of clinical decision‐making autonomy.

### Psychological burden, liability risks, and the role of “second victim syndrome” in defensive medicine

The psychological burden observed in the context of DM stems from physicians’ fear of liability, complaints, reputational damage, and the associated phenomenon known as “second victim syndrome.” First described in 2000, this concept refers to the emotional and psychological strain experienced by physicians who fear they may have caused harm to a patient – either due to actual treatment errors or complications with objectively negative outcomes.^3^, ^19^, ^20^ This phenomenon has been well documented, particularly among surgeons who witness procedure‐related complications in their patients. Such events often leave a lasting emotional impact on the surgeons themselves and may trigger a range of psychological reactions – including guilt, anxiety, heightened empathy, and burnout – as well as somatic symptoms such as sleep disturbances. As a result, affected physicians may increasingly turn to defensive medical practices to avoid similar experiences in the future.^21^

Comparable findings have been reported in urology: a survey among Spanish urologists on medical liability proceedings revealed that physicians affected by litigation suffer greatly from the consequences of criminal prosecution and, especially after having undergone such proceedings, are more likely to engage in defensive medicine.^22^

Closely related to the second victim syndrome is the concept of the “clinical judicial syndrome”, which describes the emotional and psychological distress physicians experience during professional or disciplinary proceedings – a phenomenon that remains insufficiently studied to date.^23^, ^24^ In this context, the seminal 2000 article by A.W. Wu, who articulates the emotional toll of medical error on physicians with remarkable empathy, is well worth reading.^20^

A noteworthy review article on the second victim phenomenon and the clinical judicial syndrome was published by Ozeke et al. in 2018.^25^ The authors follow the logic of the second victim model to its natural conclusion and describe a cascade of consequences that – almost inevitably – leads to defensive medical behavior. In addition to the first victim (the patient), and the second victim (the physician involved), they introduce a third victim (the institution, which may suffer reputational or financial damage) and a fourth victim (other patients, who are negatively affected by subsequent overly cautious or defensive medical decisions).^20^, ^25^, ^26^

In dermatopathology, diagnostic errors can have far‐reaching consequences – not only for the directly affected patient (first victim), but also for the responsible physician (second victim), the institution (third victim), and future patients (fourth victim). The latter may be indirectly harmed by an unconscious increase in diagnostic caution, manifesting in defensively motivated decisions – for example, the pathological upgrading of benign lesions to “questionably malignant” or the recommendation of unnecessary re‐excisions prompted by vague diagnostic wording and qualifying marginal notes. In such contexts, the use of additional immunohistochemical or molecular diagnostic methods serves less to clarify the diagnosis than to provide documented legal reassurance. While these measures signal thorough engagement with the case, they do not necessarily replace a well‐founded diagnostic judgment. This leads to a shift in focus from the core morphological expertise of the dermatopathologist toward a demonstrative logic of reassurance, a characteristic feature of defensive medicine that may be understandable but is not always in the patient's best interest.

### Structural and economic drivers of defensive medicine

From an economic perspective, DM represents a significant and often underestimated cost driver. Inadequate error management and insufficient organizational structures foster defensive medical practices – especially among younger physicians who must navigate a complex clinical environment lacking a culture of error transparency and clear procedural guidelines.^19^ As early as 2012, a survey of fourth‐year medical students and third‐year residents in Chicago showed that legal liability for medical errors is a factor that must be considered in clinical decision‐making.^27^ According to the study, 92% of the surveyed medical students and 96% of the residents reported using additional medical measures (assurance behavior; i.e., providing extra services of minimal clinical value). Moreover, 34% of the students and 43% of the residents admitted to occasionally engaging in avoidance behavior – i.e., withholding services perceived as risky or avoiding patients deemed high‐risk.^27^ However, experienced physicians are not immune to defensive practices either: personal experiences with adverse outcomes – whether directly encountered or observed in colleagues – combined with systemic pressures within clinical institutions and rising patient expectations, can drive even senior clinicians toward defensive behavior.^5^ In addition to the desire for legal protection, economic incentives may also contribute to defensive medical behavior – especially within reimbursement systems that financially reward additional diagnostic procedures, even when their clinical utility is unproven. Studies have shown that financial targets and performance‐based compensation correlate with defensive decision‐making, thereby structurally reinforcing such behavior.^19^

## DEFENSIVE DECISION‐MAKING IN DAILY DERMATOPATHOLOGY

### Diagnostic uncertainty, liability, and increasing workload – or, “is it better to be safe than sorry”?

Case law, patient autonomy and will, and economic pressures are increasingly pushing physicians of all ages and specialties into defensive behavior. From a legal perspective, there is no obligation to pursue maximum diagnostics; rather, physicians are expected to adhere to the recognized medical standard. Medical decisions must be evaluated *ex ante* – based on the information available at the time – and not *ex post*, with hindsight. Furthermore, the principle of economic efficiency under § 12 of the German Social Code Book V (SGB V) applies, which explicitly excludes unnecessary diagnostics. Although reliable data on error rates in pathology appear to be lacking, the factors for diagnostic error identified by Kostopoulou et al. in primary care can, in principle, be transferred to pathology.^28^ In general, the error rate in pathology – due to a high degree of standardization, comparable to radiology – is relatively low, at approximately 2%–5%. This contrasts with other specialties, where error rates in general or emergency medicine have been reported between 10%–15%.^28^, ^29^ However, since pathology stands at the very beginning of clinical decision‐making, missed or incorrect diagnoses can have a major impact on subsequent patient management. Thus, diagnostic errors in pathology may be rare, but they are often highly consequential.

As Vincent Burgert puts it bluntly: *“Considering that a conviction for negligent manslaughter under § 222 of the German Penal Code carries a penalty of up to 5 years' imprisonment or a fine, even a single misdiagnosis can result in significant consequences. Given the high number of cases processed daily, this is a real and serious risk.”* ^29^ From the perspective of the pathologist affected, it is irrelevant whether such an error is statistically rare – the impact is immense. Against this legal background, the writings of V. Burgert are strongly recommended to readers interested in the medico‐legal implications of diagnostic errors.^29^

But where exactly, in daily pathological practice, does the required duty of care aligned with the medical standard end – and where does pathological overdiagnosis in the sense of maximal diagnostics begin? In clinical medicine, detailed documentation is often seen as a protective measure against liability. However, this strategy appears ill‐suited for pathology. After all, who would state in a histopathological report: “Histologically unequivocal, no additional testing required”? Such a formulation would only be conceivable in diagnoses that are clearly uncritical, for example an epidermal inclusion cyst, and these rarely raise concerns about misdiagnosis. In situations where a second opinion or tumor board may be reasonable but not immediately necessary, it might instead be listed as an optional recommendation.

It is therefore not surprising that in modern medicine there is increasing pressure, especially from patients, for “more” or “further” diagnostics. Yet even among physicians, overtesting and overtreatment are often perceived as safer options than undertreatment. The term *undertreatment* already carries a negative connotation, implying that something essential has been omitted. The skin cancer screening program is a good example of the challenges involved in weighing the benefits and risks of early detection efforts. While screening may help detect skin cancer at an early stage, there is currently no clear evidence that it significantly reduces mortality. At the same time, overdiagnosis can occur – detecting changes that would never have become clinically relevant without screening.

This can lead to further investigations, biopsies, and treatments that may place a burden on patients. In addition, the combination of aggressive diagnostic practice and high patient expectations can further drive the number of investigations, even when the individual benefit is unclear. In such a scenario, it is essential to regularly evaluate whether screening programs are achieving their intended outcomes and whether the benefits are proportional to the potential downsides.^30^, ^31^

From this emerges – in addition to patient‐driven demands – a physician‐driven DM dynamic, creating a self‐perpetuating spiral of medical diagnostics without necessarily improving patient care.^32^ It is necessary and overdue that the concept of *overmedicalization* and *defensive medicine* becomes more widely recognized, and that physicians and institutions develop greater awareness and sensitivity to it.^33^, ^34^ The growing demand culture among patients contributes significantly to the escalation of diagnostic and therapeutic interventions. Many patients believe that more medicine automatically leads to better health, increasing the expectation pressure on physicians. Moreover, unrestricted access to medical information online allows patients to engage more actively with potential diagnoses, often leading them to request specific tests or treatments – regardless of any actual medical necessity. Patient advocacy groups also play a role by directing public and political attention to particular conditions, lobbying for specific diagnostic or therapeutic approaches. This exerts additional pressure on physicians to offer services that may not be clinically necessary but are difficult to deny for legal or economic reasons.

What measures are recommended to fulfil the duty of care in (dermato)pathology, to document a thorough evaluation of each case, and to anticipate the often instinctive expert judgement rendered *ex post*, in line with the adage that “hindsight is always 20/20,” a notion that holds especially true in dermatopathology? Diagnostic errors may only become evident years later, as the patient's disease progresses unfavorably. Even decades later, archived tissue samples can be reviewed and reinterpreted. Measures such as obtaining second or third opinions, consulting reference centers, intensifying special stains and immunohistochemistry, using soft diagnoses or diagnostic compromise formulations, and referencing guidelines or society recommendations can all support diagnostic diligence. However, these measures often gradually transition into defensive medical strategies. Assurance behaviors include the increased use of immunohistochemistry, obtaining second opinions, and consulting reference pathology. Avoidance behaviors involve refraining from evaluating diagnostically uncertain cases. All of these, while perhaps intended to reduce liability, carry well‐known drawbacks – not to mention the significant time and workload implications for the reporting pathologist.

### The role of defensive medicine in dermatopathology in the context of diagnostic uncertainty

Defensive medicine in dermatopathology refers to diagnostic decisions that are made primarily to mitigate legal liability – rather than being solely focused on patient welfare – or are explicitly requested by the referring clinician, such as the surgeon submitting the specimen. This includes excessive ancillary testing such as immunohistochemistry or molecular analyses, as well as cautious or non‐specific diagnoses aimed at avoiding legal consequences. Dermatopathology is among the most subjective subspecialties in pathology, characterized by high interobserver variability and numerous diagnostic grey zones. The combination of subjective interpretation, limited or inconclusive diagnostic tools, and legal pressure makes this one of the most critical and vulnerable areas within pathology. As one publication puts it: *“The medical subspecialty of dermatopathology ranks second highest in malpractice verdicts … and misdiagnosed melanoma is the most common reason for dermatopathology malpractice claims … Approximately 9% of a pathologist's 40‐year career … is spent with an open malpractice claim … Malpractice litigation has been described as a personal crisis for pathologists who are sued.”* *35*

Several critical areas in dermatopathology present ongoing diagnostic challenges, including diagnostic uncertainty, interobserver variability, and potential medico‐legal implications. While direct comparative data on error rates between dermatopathology and general pathology remain limited, it is undisputed that diagnostic complexity in this field is high – owing to the broad spectrum of dermatologic diseases and the subtlety of histologic distinctions. These diagnostic difficulties may increase the likelihood of legal disputes. Notable contributors to this vulnerability include high interobserver variability (i.e., discrepancies between dermatopathologists), the difficulty of distinguishing among benign, malignant, and borderline lesions, and the dilemma of overuse versus underuse of immunohistochemistry. Combined histologic, immunohistochemical, and molecular diagnostics are often necessary, yet even this triangulated approach does not guarantee definitive diagnostic certainty for many skin disorders. When applied excessively, such measures may represent defensive medicine – without necessarily improving diagnostic accuracy.

### Strategies for rational diagnostic decision‐making

There is no general legal obligation to routinely perform immunohistochemistry, molecular testing, or to obtain a second opinion. The assertion that no ancillary testing is required in cases with a clear histopathologic diagnosis should not be understood as a restriction but as an expression of differentiated, indication‐based diagnostics. This approach does not aim to reduce diagnostic thoroughness, but rather to avoid routine, clinically unjustified ancillary studies whose contribution to diagnostic decision‐making should be critically assessed on a case‐by‐case basis. Such an approach is fully consistent with recognized medical standards and the principle of economic efficiency as outlined in § 12 of the SGB V, which mandates that diagnostics must be appropriate, sufficient, and proportionate to the individual clinical and pathological context. In this sense, refraining from non‐indicated ancillary testing does not constitute defensive medicine, but rather a medically responsible prioritization of relevance, proportionality, and diagnostic clarity.

The decisive factor is that reporting adheres to accepted medical standards and that all decisions are transparently and traceably documented. The following key principles may guide a rational diagnostic approach:

1. *Medical standard, not maximal diagnostics*: Orientation should be based on the professional standard, derived from guidelines, literature, expert consensus, and commonly accepted dermatopathological practice. There is no absolute obligation to perform ancillary studies if the diagnosis is histopathologically unequivocal.
2. *Ex‐ante perspective*: Decisions must be judged based on the context at the time in the event of a legal dispute, what counts is the perspective at the time of diagnosis (ex ante), not retrospective reinterpretation with hindsight knowledge (ex post).
3. *Current legal developments*: There is a growing number of court rulings criticizing the absence of immunohistochemical or molecular confirmation – often triggered by expert witnesses who retrospectively express a preference for such tests. Nonetheless, the fundamental principle remains unchanged: Ancillary studies are indicated when the histologic diagnosis is uncertain, additional testing would significantly influence diagnosis or treatment, established guidelines or consensus statements recommend the use of such methods, or an interdisciplinary tumor board or expert panel explicitly recommends them.

If the dermatopathological diagnosis is histologically clear, if ancillary studies do not meaningfully refine the diagnosis, and if molecular or immunohistochemical testing does not add clinical value, then such tests should not be performed.

### Forms of defensive diagnostics: from immunohistochemistry to second opinions – the fine line between diagnostic diligence and overdiagnosis

#### Melanocytic lesions: Diagnostic challenges and the risk of misclassification

The distinction between benign nevi, atypical nevi, reactive melanocytic proliferations (e.g., actinic or postoperative), and malignant melanomas represents a well‐known diagnostic pitfall in daily dermatopathological practice. Studies have demonstrated both high interobserver and intraobserver variability in the evaluation of melanocytic lesions.^36^, ^37^, ^38^, ^39^, ^40^, ^41^ Similarly problematic is the issue of overdiagnosis vs. underdiagnosis – that is, exaggerating or downplaying melanocytic findings.^32^ Overdiagnosis may result in unnecessary excisions and psychological distress for the patient, whereas underdiagnosis can lead to missed or delayed melanoma diagnoses – with potential medico‐legal implications. Certain diagnostic entities – such as Spitz nevus vs. Spitz melanoma or melanocytoma vs. melanoma – remain challenging even with the use of molecular testing, which does not always provide definitive results.^42^, ^43^, ^44^

### Lymphoproliferative disorders

Early stages of mycosis fungoides (MF) are notoriously difficult to diagnose, as they show clinical and histopathologic overlap with chronic eczematous dermatoses. Immunohistochemistry and molecular clonality analyses are not always conclusive, and interobserver variability also plays a significant role in this diagnostic setting.^45^, ^46^, ^47^, ^48^, ^49^, ^50^ This raises the question: Should clonality analysis always be performed when MF is suspected? Cost is a relevant factor. Moreover, it is well known that clonal T‐cell receptor (TCR) gene rearrangements are not restricted to malignant conditions. Clonal populations can also be detected in benign inflammatory skin diseases, which limits the specificity of this method. While T‐cell clonality testing is an established adjunct tool in the diagnosis of mycosis fungoides,^51^ the results must always be interpreted in the context of clinical and histopathologic findings. It is not a definitive or standalone diagnostic criterion for cutaneous lymphoma.^52^, ^53^

### Additional case scenarios from daily dermatopathology

The dilemma of “borderline” cases is a well‐recognized phenomenon in dermatopathology and frequently a reason for defensive medical behavior. Entities such as Spitz nevus vs. atypical Spitz tumor vs. Spitz melanoma, dermatofibroma vs. dermatofibrosarcoma protuberans (DFSP), or cutaneous leiomyosarcoma vs. benign leiomyoma are among the best‐known examples. Other morphological pitfalls are also well‐known, especially within the spectrum of granulomatous dermatoses, where infectious etiologies must be reliably excluded – for example: Leishmaniasis vs. cutaneous sarcoidosis, granulomatous or pseudolymphomatous fungal infections vs. pseudolymphomas or lymphomas, or Borrelia infection vs. syphilis vs. cutaneous T‐cell lymphoma.^54^ Another characteristic challenge in dermatopathology is the underdiagnosis of rare tumors, such as Merkel cell carcinoma vs. metastatic small cell neoplasms, or rare cutaneous sarcomas and soft tissue tumors (e.g., PEComa, rare liposarcomas), for which even extensive immunohistochemical or molecular workup may not provide a definitive answer.

Titus et al. investigated the influence of medicolegal concerns on diagnostic behavior in the assessment of melanocytic skin lesions (MSL), and their findings are striking:^10^ Nearly one‐third of surveyed pathologists reported that legal concerns influenced them to classify melanomas more severely, and 95.2% (nearly all) practiced at least one form of defensive diagnostics. These included: seeking second opinions or consulting colleagues/specialists even when confident in the diagnosis, preparing additional histologic sections, increased use of immunohistochemical or molecular tests to objectify diagnoses, even when histology alone would likely suffice. Furthermore, many pathologists reported a tendency to err on the side of malignancy to avoid legal consequences from a potential false‐negative (benign) diagnosis. In cases where definitive histologic classification was not possible, recommendations were often made to perform rebiopsy or complete excision, to increase diagnostic certainty – even if not strictly indicated.^10^

Carney et al. published similar findings in 2016: Here too, dermatopathologists reported using the same defensive diagnostic strategies, although the study did not identify a direct correlation between actual litigation experience and the increased use of defensive measures.^55^ Key factors influencing the likelihood of defensive behavior included: years of experience as a pathologist, or previous exposure to litigation. Pathologists who had been personally sued or who had served as expert witnesses in melanoma cases were particularly prone to defensive measures. Notably, dermatopathology subspecialists demonstrated higher rates of defensive practices than general pathologists.^10^ Reisch LM addressed a related question in a 2020 publication and differentiated between liability‐driven motivation and patient safety concerns for additional testing.^35^ According to their data, 95% of dermatopathologists reported performing at least one defensive diagnostic measure due to liability concerns and 99% reported at least one such measure motivated by patient safety. This suggests that defensive medicine in dermatopathology may serve both as a mechanism of risk management and as a sincere effort to minimize diagnostic error.^35^

Interestingly, pathologists who diagnosed a large number of melanocytic lesions annually were more confident in their assessments and relied less on ancillary testing. By contrast, those with less experience in melanoma diagnostics showed higher rates of defensive practices, particularly in ordering immunohistochemistry and molecular analyses.

### Technological faith and the paradigm of diagnostic reassurance

One important aspect of defensive medical decision‐making is not solely driven by legal or organizational concerns, but is also deeply rooted in cultural attitudes. In a widely cited commentary, Leff and Finucane (2008) introduced the term “Gizmo Idolatry” – the uncritical veneration of technological procedures in modern medicine, regardless of their actual benefit to the patient.^56^ This mindset is based on the implicit assumption that technical interventions are inherently more objective, safer, and thus superior, even in situations where clinical experience and contextual judgment would be sufficient. In dermatopathology, this phenomenon is particularly evident in the use of immunohistochemical and molecular ancillary tests, which are frequently performed to confirm diagnoses that are already well established. Beyond the pursuit of diagnostic certainty, such measures often serve to demonstrate professional diligence (proof of competence) and to generate documentable objectivity (objective proof) – factors also described in the “Gizmo” debate as typical expressions of a technology‐oriented reassurance logic. The authors explicitly warn against a technologically driven medical activism, which not only generates costs and conceals diagnostic uncertainty, but often substitutes genuine clinical judgment with procedural excess.

## EXPERT CONSULTATION, SECOND OPINIONS, AND DUAL REVIEW IN (DERMATO)PATHOLOGY

Liability in the context of consultative assessments and second opinions of pathological findings is a complex issue that depends on various factors. In principle, the primary (dermato‐)pathologist remains responsible for the accuracy of their diagnosis. Analogous to expert witness liability, the following applies to dermatopathological consultations: When a colleague is consulted for a second opinion, they provide a professional assessment without automatically assuming full responsibility for the entire diagnostic report. The consulting pathologist may be held liable particularly if their assessment is erroneous and the primary reporting dermatopathologist relies on it, leading to harm to the patient. In general, liability on the part of the expert only arises in cases of gross negligence or intent. It is crucial that the consulting pathologist clarifies whether their assessment is based on limited information, for example due to missing clinical data or suboptimal material. In summary, the dermatopathologist who is primarily responsible remains generally liable for the report, especially if they adopt a second opinion without critical review. The consulting pathologist may also be held liable if their assessment was incorrect and directly contributed to patient harm. Although the consultant is not in a contractual relationship with the patient, joint liability of both pathologists may occur under tort law (e.g., bodily harm). Clear documentation and precise communication are essential to minimize liability risks for both parties.

This legal uncertainty surrounding consultations and second opinions significantly contributes to defensive medicine in dermatopathology. (Dermato‐)pathologists face the dilemma of either regularly involving consulting pathologists for legal safeguarding or assuming sole responsibility for their diagnoses – both of which carry legal risks. Since the primary pathologist remains liable for the diagnosis even when seeking an external opinion, this often leads to additional investigations, extended diagnostics, or cautious wording of reports to safeguard against potential misdiagnoses. At the same time, consulting pathologists face the risk of shared liability, especially when providing incorrect or ambiguous assessments.

In this context, a clear distinction must be made between different types of consultative and reference assessments, as they entail different legal consequences and must be evaluated accordingly. Reference is made here to the recommendations on consultations and second opinions issued by the *German Association of Pathologists* (BDP) and the *German Society of Pathology* (DGP) on 03.09.2010. ^57^ These recommendations have high practical relevance, as they provide pathologists with a structured approach to consultations and second‐opinion procedures. However, they also highlight challenges in funding and liability, especially for second opinions requested by patients and for telepathological consultations. These recommendations are not binding but intended to serve as guidance. The legal and liability‐related aspects of consultative and second‐opinion diagnostics are extremely complex, often difficult, or impossible to grasp at first glance for non‐lawyers, and are subject to differing frameworks. Interested readers are referred to the abovementioned BDP position paper.^57^ It provides guidance on organizational procedures, responsibilities, and billing modalities, and can serve as a helpful reference for the practical handling of consultations and second opinions in pathology.

This position paper distinguishes between different forms of pathological consultation:^56^

1. Primary pathologist seeks their own diagnostic advice from a consulting pathologist, with or without additional required ancillary testing on the material: this describes the practice of consultative assessments as discussed in this manuscript. The primary pathologist seeks the opinion of another pathologist, without any request from the patient or submitting physician, and asks for completion and/or refinement of the diagnosis.
2. Treating physician or patient requests a second opinion: in this case, the diagnosis already issued is reviewed. Since the German Uniform Value Scale (Einheitlicher Bewertungsmaßstab, EBM) and the German Medical Fee Schedule (Gebührenordnung für Ärzte, GOÄ) typically do not provide for legal entitlement to a second opinion in the field of pathology, funding is often unclear.
3. Study‐related consultations: pathological consultations within the framework of scientific studies play an important role in quality assurance. In such cases, the study sponsor must cover the costs of the consultative pathology and material shipment.
4. Telepathological consultation: the recommendations do not yet provide definitive regulations for telepathology, but they point to additional liability issues, especially in international consultations.

The terms consultation, second opinion, and four‐eyes principle are frequently used in medical diagnostics, but they have different meanings, purposes, and legal implications. Especially in pathology and dermatopathology – where diagnostic uncertainty plays a major role – it is essential to distinguish clearly between these terms. A consultation is a professional advisory opinion or additional co‐assessment by another specialist to clarify a differential diagnostic question or to obtain an additional diagnostic perspective. A second opinion is an independent, autonomous re‐evaluation of an already issued finding by a second specialist. The four‐eyes principle refers to a systematic dual review by two physicians who check each other's work before the final release of the report.

Particularly in dermatopathology – where reports are often influenced by subjective judgments and diagnostic grey areas – all three concepts can play an important role. A clear distinction between these terms is especially important in the context of liability, to avoid misunderstandings and to define responsibilities clearly.

## DISTINGUISHING DEFENSIVE MEDICINE FROM DERMATOPATHOLOGICAL DIFFERENTIAL DIAGNOSIS

Clearly defined situations and diagnostic recommendations from oncological guidelines and expert panels must be strictly distinguished from defensive measures in dermatopathology. In such situations, an expanded diagnostic approach is routinely required to ensure accurate diagnosis – these are measures to fulfil the duty of care by making the most accurate diagnosis possible based on the available biopsy or specimen. In routine dermatopathological melanoma diagnostics, immunohistochemical stains are regularly performed in the following contexts and are not to be considered acts of defensive medicine *sui generis* in all cases, but rather as an essential part of diligent and guideline‐compliant diagnostics due to the specific morphological nature of melanomas:^58^ Immunohistochemical panel testing using various melanocytic differentiation markers with differing sensitivities and specificities is employed for the primary diagnosis of the tumor and for the differential diagnosis of other entities. These tests are used to confirm the dignity of melanocytic tumors and to distinguish between benign lesions and melanoma simulators. They also permit detection of microinvasive tumor cells and allow precise measurement of vertical tumor thickness according to Breslow. Furthermore, they are crucial for identifying perineural sheath invasion/infiltration and for evaluating lesions suspected to be metastatic. In the context of initial staging in primary melanoma, they are essential for the evaluation of sentinel lymph nodes.

Additionally, they are employed to assess the tumor‐free status of surgical margins in excisional specimens, especially in chronically sun‐exposed skin.^58^ This also applies to immunohistochemical assessment of melanocytic lesions in specific anatomical sites, such as the palmoplantar and facial regions, where these stains are urgently required to visualize subtle findings on hematoxylin and eosin‐stained sections and to properly interpret features that often appear discordant with clinical impressions.^59^

Likewise, an intensified tissue workup in the context of so‐called “invisible dermatoses” does not constitute defensive medicine *sui generis*. The term “invisible dermatosis” can be used both in clinical and histopathological diagnostics. It refers to dermatoses that show subtle histological alterations despite being clinically visible and morphologically identifiable.^60^, ^61^ These cases represent a diagnostic challenge for general pathologists and dermatopathologists alike. It is essential to understand that minimal changes in a skin biopsy do not necessarily indicate absence of disease or merely nonspecific findings.^61^ These biopsies are diagnostically demanding, especially for general pathologists, as histopathological alterations are often subtle or difficult to identify. However, an experienced dermatopathologist can provide a specific diagnosis in more than 50% of cases by identifying subtle histological features and applying specialized knowledge.^61^

It is also not a form of defensive medicine when dermatopathologists find themselves in a kind of “diagnostic self‐defense situation,” for example when extremely small biopsies are submitted together with extensive diagnostic questions. Inadequate sample quality, such as superficial or fragmented biopsies, is a common issue in dermatopathology. Well‐known examples include nail plate biopsies submitted with the question of subungual melanoma; tiny superficial shave biopsies submitted to rule out Lentigo maligna; or superficial biopsies submitted with a suspicion of panniculitis, even though there is clearly no attached fat tissue in the specimen. In such cases, there is a major discrepancy between the submitted material and the diagnostic expectations or requirements from the referring clinical colleagues.^62^ As Wolfgang Weyers has pointed out, inadequately representative biopsies – such as superficial shaves or overly small punch biopsies – can severely limit diagnostic accuracy and may promote overdiagnosis, not out of uncertainty, but as a result of structural insufficiency.^63^ In such cases, a reliable histopathological assessment is not impossible because the (dermato‐)pathologist is unwilling to provide a diagnosis, but simply because there is insufficient processable, and assessable tissue material. Any corresponding limiting wording in the report is therefore not an expression of defensive medicine but a necessary safeguard against unrealistic expectations from referring physicians.

Similarly, the absence of clinical information complicates interpretation of histological findings. To avoid misdiagnoses, close collaboration between clinicians and pathologists – as well as photographic documentation of the clinical presentation, as consistently as possible – is indispensable. This applies equally to the diagnosis of inflammatory dermatoses and melanocytic neoplasms.^62^, ^63^, ^64^

## HOW CAN THE PHENOMENON OF DEFENSIVE MEDICINE BE APPROPRIATELY MANAGED IN EVERYDAY DERMATOPATHOLOGICAL PRACTICE?

How should this concept of defensive medicine be addressed amid the exhausting daily diagnostic routine? How is a balanced diagnostic approach possible?

An appropriate handling of defensive medicine in dermatopathology requires a balance between diagnostic diligence, economic feasibility, and medical necessity. There are hardly any fixed guidelines. This final paragraph, which reflects a personal perspective and is not intended as a binding directive, highlights possible strategies for dealing with defensive practices in dermatopathology.

In general, a healthy level of composure is advisable in the day‐to‐day professional routine of dermatopathology. The knowledge that in a multitude of studies – as previously mentioned –fear of liability and the associated defensive measures tend to decrease with the pathologist's age and experience can be reassuring. Where available, standardized diagnostic approaches should be applied, and clear criteria as well as evidence‐based guidelines (e.g., WHO classification, S3 guidelines) can be used to support objective diagnostic decision‐making. At the same time, such guidelines should be critically reflected upon, as they may themselves contain potentially defensive formulations: for example, the WHO classification of skin tumors attributes a “minimal potential for malignant transformation” to lentigo simplex and classifies dysplastic nevi as a “biological intermediate” between benign nevi and melanomas.^65^ Such statements may be scientifically well‐founded but carry the risk of promoting overdiagnosis and an expansion of indications in practice – not out of diagnostic necessity, but for legal precaution. Where applicable, structured diagnostic algorithms should therefore be used.

Clinicopathological correlation must be urgently strengthened through close communication with referring clinicians to clarify unclear cases before additional analyses are requested. In this way, the use of supplementary investigations can be guided by the clinical question rather than purely legal considerations. Knowledge of relevant clinical information (e.g., previous findings, dermatoscopy, clinical course) is essential to minimize diagnostic uncertainty. Transparency and clear diagnostic phrasing should be preferred. It appears important to formulate precise and unequivocal diagnoses rather than vague findings with unnecessary caveats in order to avoid legal ambiguity. If a definitive diagnosis is not possible, the diagnostic limitations should be clearly stated rather than softened by legally motivated relativizations. A conscious, evidence‐based diagnostic approach – taking into account clinical relevance, standardized criteria, and interdisciplinary communication – helps minimize defensive medicine in dermatopathology. (Dermato)pathologists should distinguish between meaningful diagnostic safeguarding and unnecessary overdiagnosis to protect both patients and themselves as effectively as possible.

## APPROACHES TO A RATIONAL DIAGNOSTIC

### Metacognitive processes in dermatopathological diagnosis

An often‐overlooked factor that can influence the histopathological assessment of challenging dermatopathological cases is the concept of metacognition. This cognitive process encompasses the self‐awareness and reflection of one's own thinking, understanding, and diagnostic decision‐making processes.^58^, ^66^ The term metacognition was first introduced in 1979 by American developmental psychologist John H. Flavell and refers to the knowledge and regulation of one's own cognitive processes. His work laid the foundation for numerous subsequent studies in the field of cognitive developmental psychology.^67^ A more modern definition describes metacognition as thinking about one's own thinking and includes the ability to reflect on, control, and optimize cognitive processes. Metacognitive knowledge and control in the sense of self‐regulation are typically grouped together: knowledge about one's own cognitive capabilities, understanding of which strategies are helpful in particular situations, and awareness of one's own cognitive errors and biases constitute metacognitive knowledge. Strategies for problem‐solving, adaptation of those strategies, and self‐reflection are observed as part of metacognitive control. Awareness that both clinical and histopathologic diagnoses may involve uncertainty – particularly in the evaluation of complex melanocytic lesions – can encourage pathologists to critically examine their own thought processes. This may lead to more conscious self‐monitoring of diagnostic reasoning and result in the deliberate initiation of ancillary testing or the pursuit of a second opinion.^66^

Currently, no specific studies exist that directly examine the relationship between metacognition and defensive medicine. However, it can be theoretically assumed that a well‐developed metacognitive competence – that is, conscious reflection on one's own thinking and decision‐making – may help reduce defensive medical practices. The targeted promotion of metacognitive skills could support dermatopathologists in more consciously evaluating their diagnostic processes, recognizing cognitive biases, and making better‐informed, patient‐centered decisions – independent of liability‐related concerns. Although empirical evidence for this relationship is still lacking, training in metacognitive strategies within the clinical setting may, over time, contribute to a reduction in defensive diagnostic behavior.

### Digital pathology / artificial intelligence and automation: relief or new source of error?

Is digital pathology and artificial intelligence (AI) a promising tool to counteract excessive defensive practices in dermatopathology? Digital pathology and the use of AI are considered promising approaches to improve diagnostic accuracy in dermatopathology.^68^ Especially in the context of defensive medical practices, the question arises whether these technologies can help reduce diagnostic uncertainty and thereby limit overdiagnosis or unnecessary additional testing.

A systematic review and meta‐analysis published in 2023 by McGenity et al. assessed the diagnostic accuracy of AI in analyzing digital pathology images.^69^ The results showed an average sensitivity of 96.3% and specificity of 93.3%, indicating high diagnostic accuracy of AI systems in this area. However, the authors also reported significant heterogeneity in the study designs of the included publications. Details regarding case selection, data splitting for model development and validation, as well as raw performance data, were often either incomplete or missing entirely. While AI was shown to have high diagnostic accuracy in the reviewed areas, a more rigorous and precise evaluation of its actual performance is still necessary.^69^

Another example of successful AI application in dermatopathology is a deep learning system developed and tested for the detection of sentinel lymph node metastases (NM) in melanoma and for distinguishing nodal nevi (INN) in digital whole‐slide images. In the study by Siarov et al., which included 485 digitized specimens, the model achieved a performance comparable to that of experienced dermatopathologists.^70^ While the AI system achieved high sensitivity in detecting metastases, differentiating between nodal nevi and melanoma metastases was more difficult – likely due to morphological similarities and limited training data. The results underscore AI's potential to reduce interobserver variability and to help standardize diagnostics, although further validation studies are needed before broad clinical implementation can be recommended.^70^ Digital systems and AI‐based algorithms may help reduce inter‐ and intraobserver variability by providing standardized and reproducible analyses. This could be particularly helpful in assessing melanocytic lesions, which are often associated with high diagnostic uncertainty. Convolutional neural networks (CNNs) have shown high accuracy in distinguishing melanomas from nevi and can aid in detecting mitotic figures, tumor margins, and immunohistochemical markers.^71^ Initial models for the classification of cutaneous lymphomas and reactive infiltrates are already achieving diagnostic performance comparable to that of experienced dermatopathologists. Despite these advances, challenges remain regarding interobserver variability, data quality, as well as ethical and liability‐related issues. AI can improve the reproducibility and efficiency of dermatopathological diagnostics, but it requires careful validation and thoughtful integration into clinical workflows. It should be seen as a valuable assistive tool, not a replacement for the clinical expertise of the pathologist.^71^ Against the backdrop of the question whether AI can reduce or even prevent defensive medical practices, no definitive answer can currently be given – although the authors would tend to say “no” at this time. Developments in this field are highly dynamic. While AI presents promising tools to support diagnostic workflows, uncertainties remain concerning its practical implementation, reliability, and legal implications. At this stage, a final assessment is not feasible, as both the technology and its applications in medicine are evolving rapidly.

The idea that AI might eventually replace the classical four‐eyes principle in seemingly clear‐cut cases is paradoxical: in unequivocal cases, there is *per se* no need for a second opinion. In contrast, in complex cases, there is currently no legal basis to justify relying solely on AI. Such systems therefore remain without a solid legal foundation – pure future speculation. One central aspect of integrating AI into dermatopathology revolves around legal liability in the event of a diagnostic error. It remains unclear who bears responsibility if an AI‐assisted diagnosis turns out to be wrong. Is the (dermato‐)pathologist still fully liable despite the use of AI? Or could part of the responsibility shift to the provider of the AI system? Has the technology been sufficiently validated, or is it a legally risky experimental method? These fundamental questions remain unresolved and are likely to delay the widespread adoption of these technologies. There are currently few court decisions addressing liability in the context of AI‐assisted diagnoses. Without clear legal precedents, the fear of litigation remains high, which in turn further fuels defensive medical behavior. The current uncertainties in liability law and the technical limitations of AI, in the authors' view, clearly reinforce defensive tendencies in dermatopathology. This is supported by the observation that the legal framework for using AI in medicine remains unclear, and that no existing regulatory model currently grants AI‐assisted decisions any liability‐reducing effect.^72^

To ensure that AI in dermatopathology is established not only as a technological innovation but also as a reliable diagnostic tool, further research, ongoing validation, and clear legal frameworks are essential. Only through targeted development, standardized implementation, and trust‐building measures can AI truly fulfil its potential – not only to increase efficiency, but also to improve diagnostic certainty without generating new uncertainties or reinforcing defensive reflexes.

## CONCLUSIONS AND OUTLOOK

Defensive medicine is a widespread and multifaceted phenomenon in dermatopathology. It manifests, among other things, in the form of additional immunohistochemical or molecular analyses, cautious wording of reports, or the solicitation of second opinions – particularly in diagnostically challenging cases. Various influencing factors – medical, psychological, legal, and structural – can be identified, acting either individually or in combination. Numerous aspects contribute to the tendency toward diagnostic safeguarding, including variability in diagnostic interpretations, uncertainty regarding rare entities, and the tension between due diligence and economic pressure. Guidelines and tumor boards can provide orientation in this context but may also lead to a shifting of responsibility. A clear distinction between necessary differential diagnostics and overcautious safeguarding is often difficult to draw in individual cases.

In the long term, a differentiated approach to indications, the targeted use of ancillary tests, and the strengthening of clinicopathological communication appear to be central elements in reconciling diagnostic quality with resource efficiency. Subspecialization, structured decision‐making algorithms, and transparent legal frameworks can help reduce uncertainty and appropriately address diagnostic complexity. Dermatopathology exemplifies many subspecialized areas of dermatology in which diagnostic uncertainty, high expectations, and legal pressure intersect – defensive medicine is particularly visible and analyzable here, making it highly relevant to other subspecialties as well. Neither legal guardrails nor institutional routines can relieve physicians of their professional responsibility – on the contrary, systemic pressures demand careful balancing between legal precaution and medical proportionality. For the individual dermatopathologist, the situation in everyday practice often presents an almost insoluble dilemma. The range extends from seemingly banal routine specimens – often submitted without meaningful clinical information, photo documentation, or precise diagnostic suspicion – to decision‐critical cases with immediate therapeutic implications for the patient. In this reality, defensive diagnostics are often less an expression of excessive caution and more a pragmatic response to structural deficiencies and insufficient information. Those working outside a highly integrated, subspecialized center – where specimen collection, clinical evaluation, and histopathological reporting are closely interlinked – find themselves in a field of constant uncertainty. In reference laboratories without direct access to imaging, prior findings, or immediate clinical feedback, the risk of overlooking a relevant diagnosis is objectively higher. The result is a safeguarding behavior that manifests in serial and step sections, immunohistochemical panels, molecular ancillary studies, and cautiously balanced report wording. Furthermore, the dermatopathologist is human. Economic interests, limited time resources, and psychological burdens must be acknowledged without being misinterpreted as unethical behavior. At the end of a complex workday, when faced with potential legal liability, there is often no institutional support. Those who can demonstrate that they did not remain passive but took “every reasonably justifiable measure” at least protect themselves from the accusation of gross diagnostic negligence – even if the diagnosis later turns out to be incorrect. In this way, despite all criticism, defensive medicine becomes a protective mechanism within a system that emphasizes individual responsibility without reliably providing structural safeguards.

What is described here, using dermatopathology as an example, similarly applies to all clinicians working in highly specialized fields of dermatology and beyond. The structural pressure to reconcile patient autonomy, legal safeguarding, economic interests, and incomplete information is not a niche phenomenon. And this will not fundamentally change in the age of artificial intelligence, automated diagnostics, or Dr. Google: in case of doubt, it is not the system that is liable – but the individual physician.

Medical decisions are never made with the knowledge of tomorrow – but under the concrete uncertainties of today.

## CONFLICT OF INTEREST STAEMENT

None.
